# Biopsy and histologic findings of the dura mater at the level of the foramen magnum in 121 CKCS with Chiari-like malformation

**DOI:** 10.3389/fvets.2022.954092

**Published:** 2022-09-07

**Authors:** Jaclyn P. Holdsworth, Dominic J. Marino, Catherine A. Loughin, Andrew D. Miller, Joseph J. Sackman, Martin L. Lesser, Marissa O'Donnell

**Affiliations:** ^1^Department of Surgery and the Canine Chiari Institute, Long Island Veterinary Specialists, Plainview, NY, United States; ^2^Biomedical Sciences, Section of Anatomic Pathology, College of Veterinary Medicine, Cornell University, Ithaca, NY, United States; ^3^Biostatistics Unit, Feinstein Institute for Medical Research, Northwell Health, New York, NY, United States

**Keywords:** dural biopsy, dura histopathology, Chiari-like malformation, syringomyelia, foramen magnum decompression

## Abstract

To describe histopathologic features found in dural biopsies of Cavalier King Charles Spaniels (CKCS) with Chiari-like malformation (CM) and identify any associations between age, duration of clinical signs, syrinx location or syringomyelia (SM, and quality of life (QOL). The medical records of 121 consecutive client owned CKCS with CM and SM, confirmed by whole body magnetic resonance imaging (MRI), that underwent foramen magnum decompression (FMD) with cranioplasty and durectomy with biopsy from 2006 to 2016 were retrospectively reviewed. Dural biopsies were submitted to a board-certified veterinary pathologist for histopathologic interpretation. The chi-square test was used to analyze associations between histologic findings and categorical variables. For continuous measures, the Kruskal–Wallis non-parametric test was used to compare distributions across pathology categories. A result was considered statistically significant at the *p* < 0.05 level of significance. The mean age, duration of pre-surgical clinical signs, and pre-operative QOL (1–5 scale) were 44.27 months, 44.78 weeks, and 2.72, respectively. Syringomyelia was found in the cervical region only in 39 of 121 (32.23%) of dogs, in the cervical and thoracic region only in 17 of 121 (14.05%) of dogs, and in the cervical, thoracic, and lumbar region combined in 65 of 121 (53.72%) of dogs. Sixty-six of one hundred twenty-one (54.55%) dural biopsy specimens had histopathology changes; fifty-five (45.45%) did not. Forty-three of one hundred twenty-one (35.54%) dural biopsy specimens had osseous metaplasia, 16 of 121 (13.22%) had evidence of fibrosis, 4 of 121 (3.31%) had arachnoid hyperplasia, and 3 of 121 (2.48%) had evidence of mineralization. Most dogs with CM were found to have histopathologic changes in the dura at the time of FMD cranioplasty was performed. These dural changes can be observed in dogs experiencing clinical signs for a time period as short as 4 weeks prior to presentation. The histopathologic changes were not associated with age, breed, duration of clinical signs, the location of syringomyelia or QOL. The influence of histopathologic changes on long-term prognosis in dogs without dural decompression is unknown since all dogs in this study had dural resection.

## Introduction

Chiari-like malformation (CM) has been described in small breed dogs and is especially common among Cavalier King Charles Spaniels (CKCS) (1–7). Most dogs with CM have syringomyelia (SM) present on imaging at the time of diagnosis (8–19). Syringomyelia is viewed as a secondary condition, thought to evolve from alteration in cerebrospinal fluid (CSF) flow secondary to chronic malformation at the foramen magnum (10–12) and brachycephaly. Presenting clinical signs of CM with or without SM, include excessive scratching of the head and neck, air scratching, cervical guarding and pain, and sometimes vestibulocerebellar dysfunction (1, 15, 18, 20–24).

In addition to osseous compression, thickening of the dura mater at the craniocervical region has been reported in human Chiari I malformation (CM-I) (25–29) and in both CKCS and non-CKCS breeds (1, 30). Removal of the outer layer of the dura mater (also termed the “dural band”) or duraplasty has been reported as an adjunctive decompression technique for human patients with CM-I. Additionally, there have been several studies describing boney decompression alone vs. boney decompression with duraplasty (26, 31–38). Boney decompression with duraplasty has been associated with a significant reduction in the volume of syrinxes with subsequent improvement of neurologic clinical signs in the human patient (36). FMD with concurrent cranioplasty and full-thickness removal of the dura called “durectomy” has been reported as a method of decompression in dogs with CM; with most dogs improving clinically with an overall improvement in QOL score (1).

Although thickening of the dura mater in humans with CM-I has been described, there are only three papers in the human literature discussing the histopathologic changes seen in the dura mater (27, 39, 40). Gross dural thickening has been described in dogs with CM (15, 20, 30), but no reports of histopathologic findings have been described in the veterinary literature.

The purpose of this study is to describe the histopathologic changes of surgically resected dura in dogs with CM and to report any association between age, the location of syringomyelia, the duration of clinical signs prior to surgical intervention, and the quality of life (QOL) pre-operatively. We hypothesized that dogs with increased age, increased duration of clinical signs, and the presence of a syrinx in the cervical, thoracic, and lumbar regions combined will be associated with more severe histopathologic changes.

## Materials and methods

### Patient population

One hundred twenty-one consecutive client-owned CKCS from 2006 to 2016 with CM and SM confirmed by whole body MRI that underwent FMD with cranioplasty and durectomy (41) were included in the study. Dogs were diagnosed with CM if both of the following criteria were evident on midsagittal MRI: the caudal aspect of the cerebellum was compressed by the occipital bone and there was elimination of the dorsal subarachnoid space at the craniocervical junction (42). Informed written consent was obtained from all owners of the animals in this study. Dogs with other known craniocervical junction abnormalities were excluded from the current study.

The medical records were retrospectively reviewed. Age, sex, duration of pre-surgical clinical signs, pre-operative QOL, the location of a syrinx, and dura mater histopathology were recorded for all patients.

### Preoperative quality of life

Prior to surgery owners were asked to complete a QOL questionnaire. This questionnaire and its components were used in a prior study published in 2007 (1). Questions related to the pet's quality of life before surgery using a scoring system from 1 to 5. The scoring system was as follows; 1 = extremely poor, considering euthanasia; 2 = poor, somewhat manageable with medical therapy; 3 = fair - does well overall; 4 = good–minor signs of disease [e.g., occasional scratching, occasionally appears painful]; 5 = excellent–minimal signs of disease [e.g., rarely scratches or appears painful]). The questionnaire also had owners report the level of disease progression prior to pursuing surgical intervention. Progression was categorized as follows: rapidly progressing (i.e., worsening weekly); progressing moderately (i.e., worsening monthly); slowly progressing (i.e., worsening about every 6 months); static/non-progressive (pursued surgery to prevent progression); or improved with medical therapy (pursued surgery to prevent progression) (1).

### Anesthetic protocol

Anesthesia entailed premedication with hydromorphone (0.1 mg/kg subcutaneously) and with atropine (0.02–0.04 mg/kg subcutaneously). Induction was performed with propofol (3–6 mg/kg intravenously), and maintenance with isoflurane in oxygen. Methyl-Prednisolone sodium succinate (30 mg/kg intravenously) and Mannitol (0.5 g/kg intravenously over 10–15 min) were also administered at the time of induction of general anesthesia. Cefazolin (22 mg/kg intravenously) was administered at the onset of surgery, and every 2 h during the surgical procedure until its completion (1).

### Surgery–FMD with cranioplasty

As previously described (42), a dorsal midline approach to the caudal occipital region was performed. A FMD with C1 dorsal laminectomy was performed using a combination of high-speed pneumatic drill and rongeurs. The rostral extent of the boney decompression was midway between the occipital protuberance and the dorsal aspect of the foramen magnum. The caudal aspect of the boney decompression includes a portion of the dorsal arch of C1 between each of the lateral vertebral foramen from the most cranial and caudal extent including the insertion of the dorsal atlanto-axial ligament. No cervical instability was noted; therefore, no additional stabilization was necessary. The lateral limits of the boney decompression were the lateral foramina of the C1 and each atlanto-occipital joint (42, 43). A full thickness durectomy was performed along the aforementioned borders of the boney decompression using a blunt hook nerve root retractor and number 11 blade. The full thickness dural sample were submitted for histopathologic review. Any arachnoid veils or adhesions present between the caudal part of the cerebellar vermis and the dorsal part of the brainstem were broken down *via* a small blunt-tipped nerve root retractor. A cranioplasty was performed utilizing a titanium mesh covered by a thin layer of polymethylmethacrylate, anchored on 5 titanium screws placed around the widened foramen magnum. A fat graft was placed over the caudal part of C1 (between the caudal part of the cranioplasty/mesh and the cranial part of C2) (42). The incision was closed routinely in three layers. Samples of dura were placed in 10% neutral buffered formalin and submitted for histopathologic interpretation by a board-certified veterinary pathologist. Samples were trimmed and paraffin embedded. Five micrometer sections cut with a microtome were transferred to slides and stained with hematoxylin and eosin (H and E). All dogs included in this study had surgery performed at Long Island Veterinary Specialists by a neurosurgical team led by one of the authors (DM).

### Histopathologic classifications

All surgically resected dural samples were evaluated and interpreted by the same board-certified veterinary pathologist (ADM). Five classification groups were established based on the major histologic change noted in the surgically resected dural samples (no evidence of inflammation or neoplasia, arachnothelial hyperplasia, dural fibrosis, dural mineralization, and dural osseous metaplasia). Distinct descriptions of each dural classification are as follows:

#### No evidence of inflammation or neoplasia

**Figure 1:**
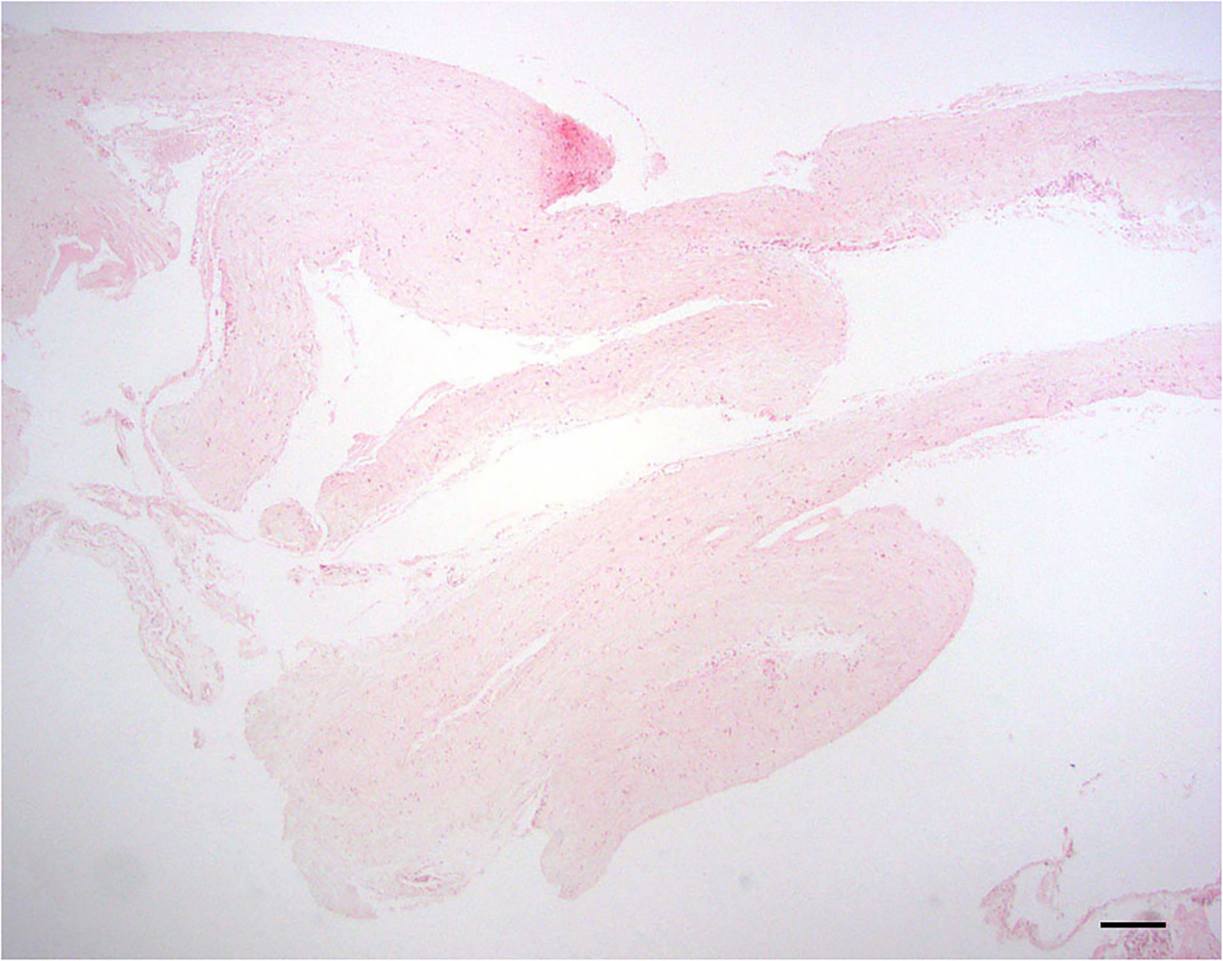
Normal dura; 10 × –Scale bar = 100 microns.

Examined sections of dura mater showed no evidence of inflammation or neoplasia. The samples consisted of normal dense, parallel streams of cellular collagen with small amounts of fibrofatty connective tissue (Figure 1).

#### Arachnothelial hyperplasia

**Figure 2:**
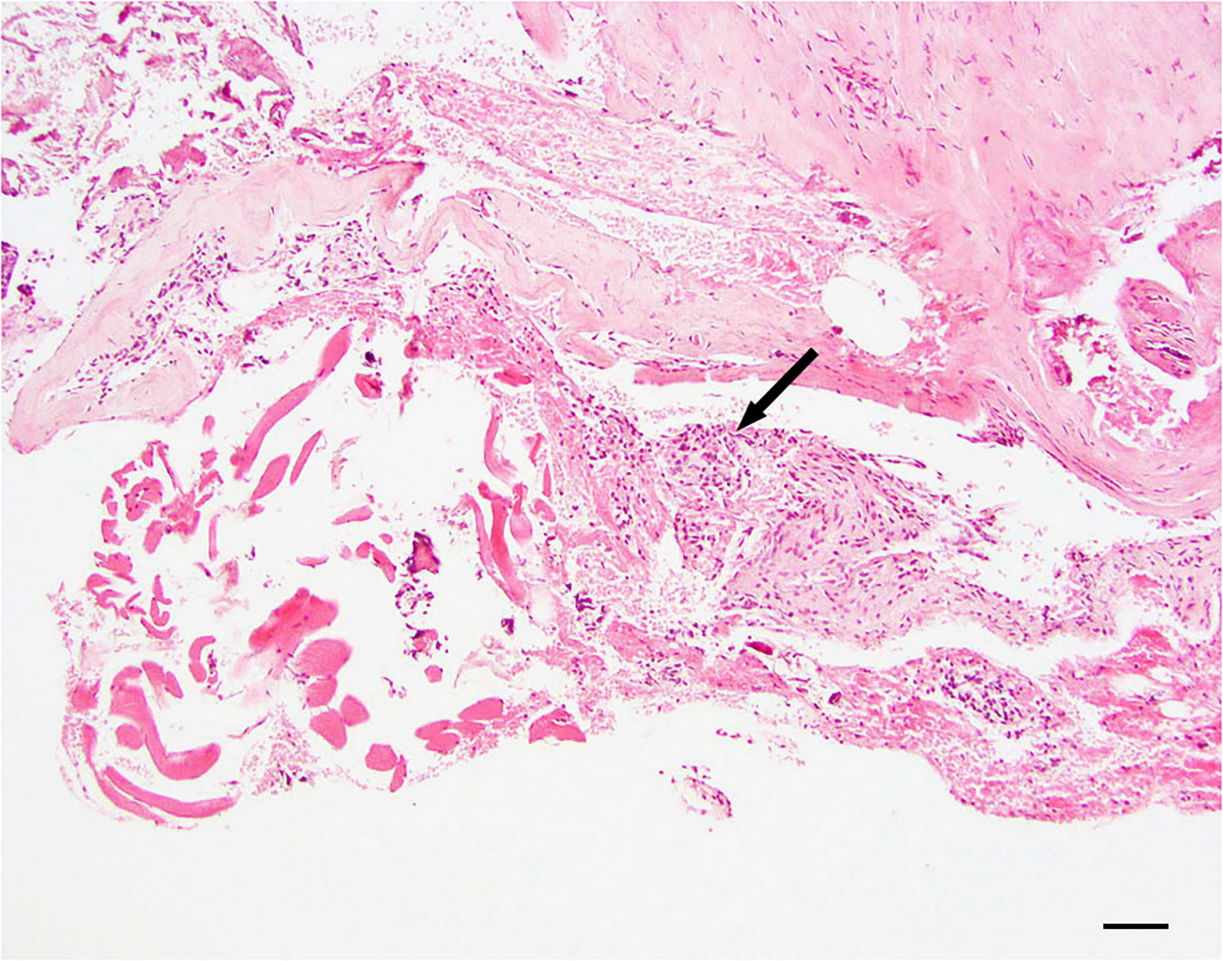
Dural arachnoid hyperplasia; 20 × –Scale bar = 50 microns; Arrow indicates arachnoid hyperplasia.

Dura samples examined showed multiple foci of hyperplastic arachnoid cells lining the inner surface of the dura (Figure 2).

#### Dural fibrosis

**Figure 3:**
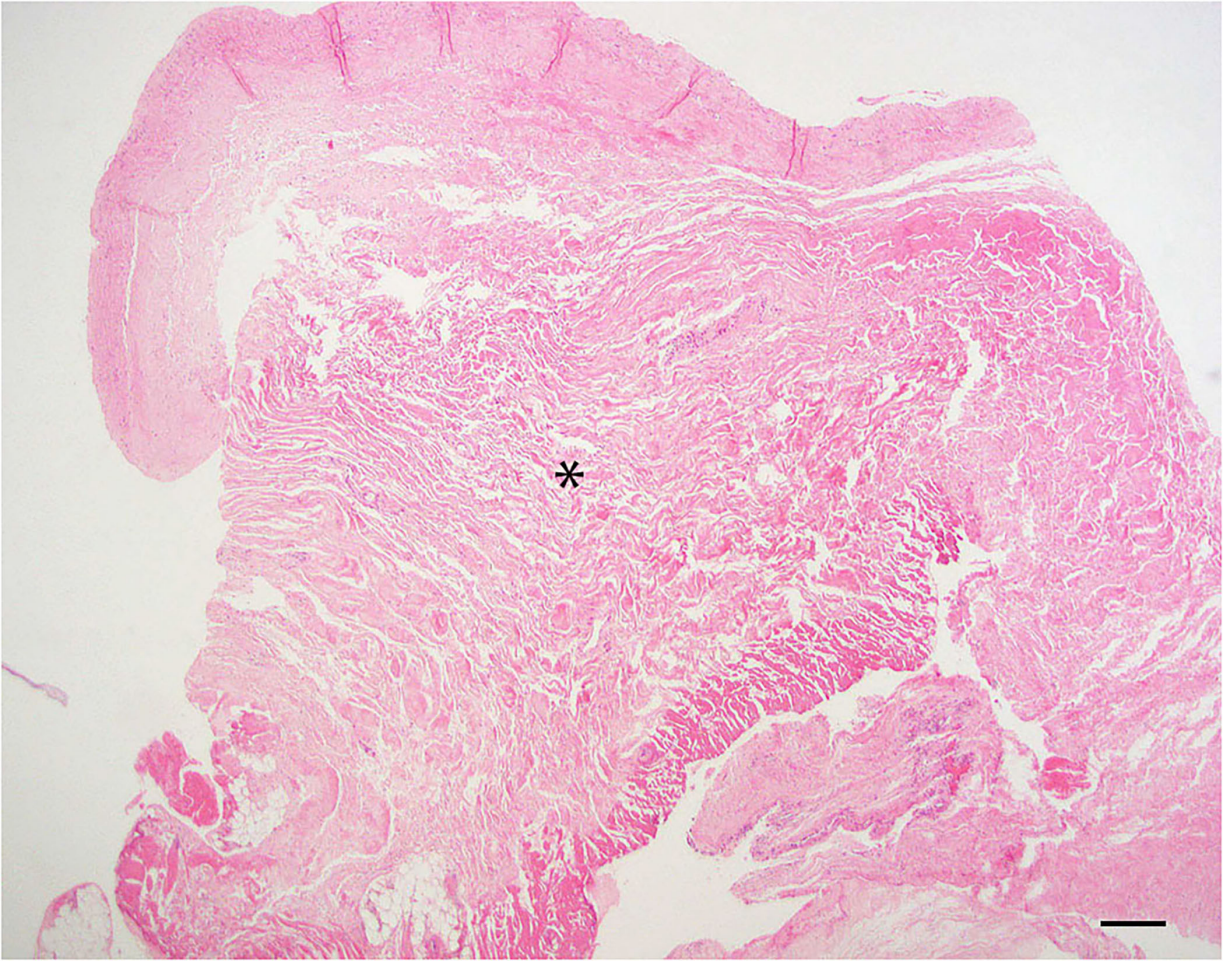
Dural fibrosis; 20× –Scale bar = 50 microns; Asterisk indicates area of fibrosis.

Dura samples examined showed thickened by hyalinized collagenous tissue of low cellularity within the specimen. The normal thickness of the dura has been distorted due to a moderate expansion of fibrous connective tissue (Figure 3).

#### Dural mineralization

**Figure 4:**
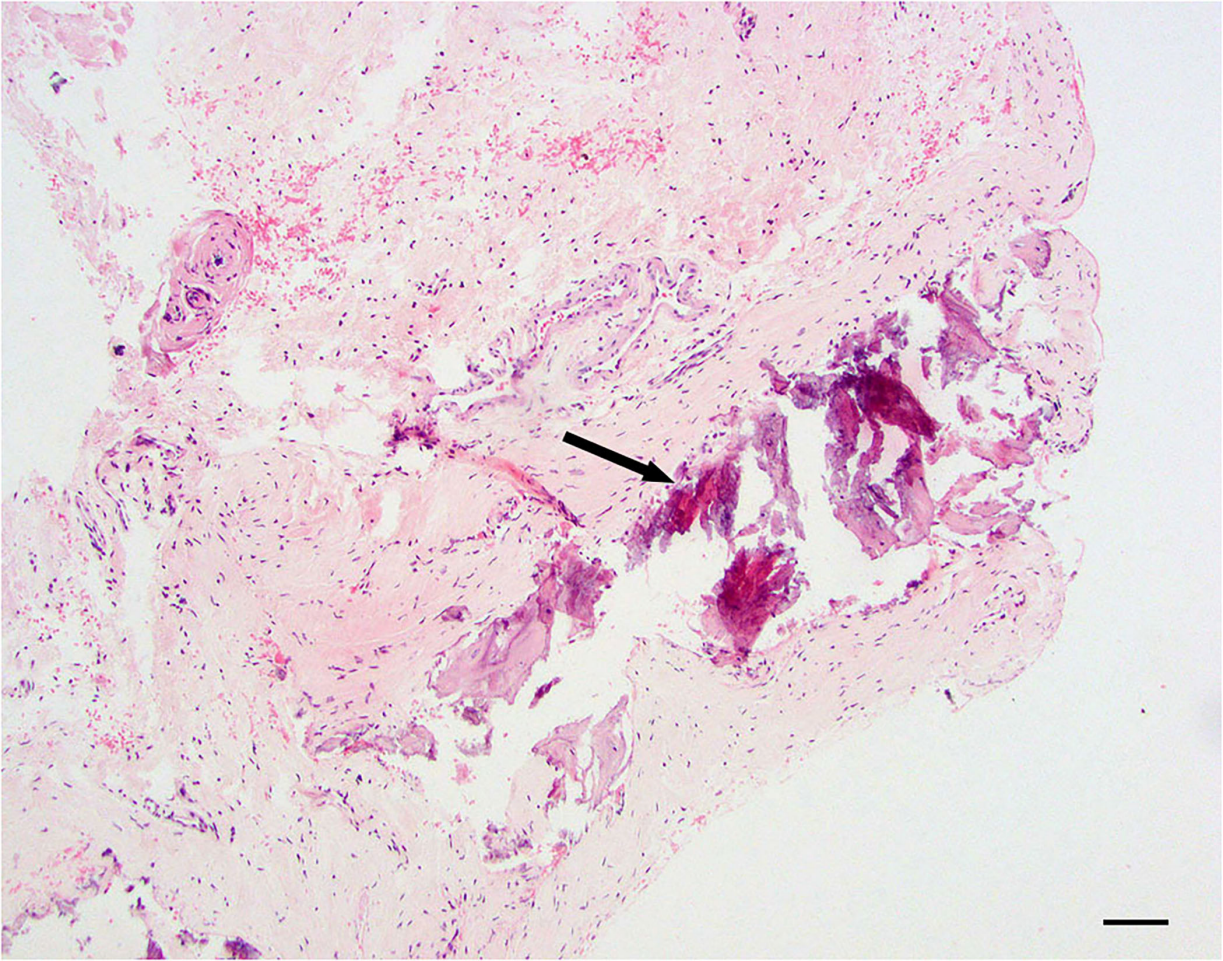
Dural mineralization; 20 × –Scale bar = 50 microns; Arrow indicated mineralization.

Dural mineralization is defined as mild to moderate, multifocal mineralized, and basophilic material within the dura mater. Collagen fibers are usually degenerative and still have some level of inflammation present (Figure 4).

#### Dural osseous metaplasia

**Figure 5:**
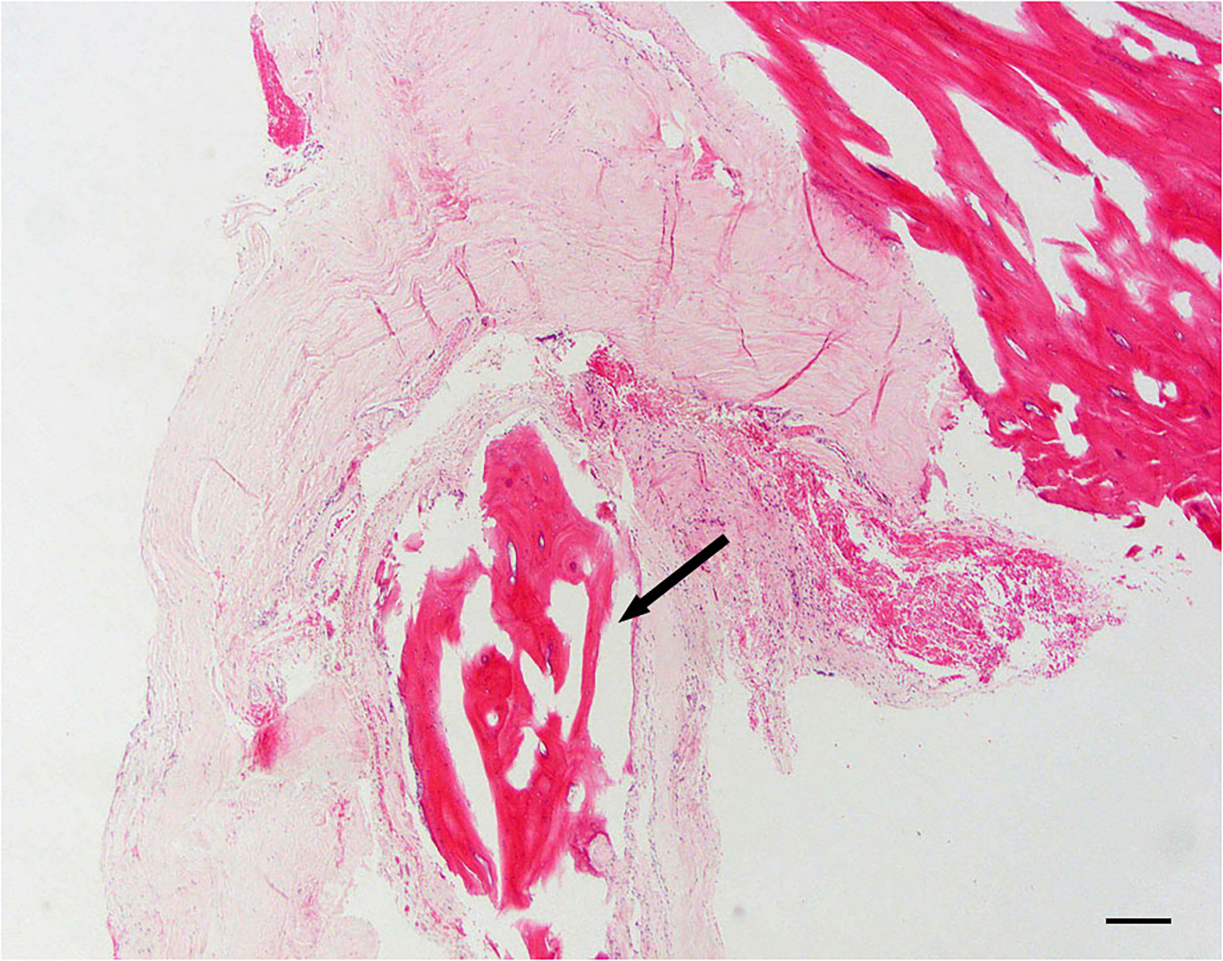
Dural osseous metaplasia; 10 × –Scale bar = 100 microns; Arrow indicates osseous metaplasia.

Dural ossification is defined as foci of osseous metaplasia embedded within the normal connective tissue of the dura mater (Figure 5).

#### Control specimens

**Figure 6:**
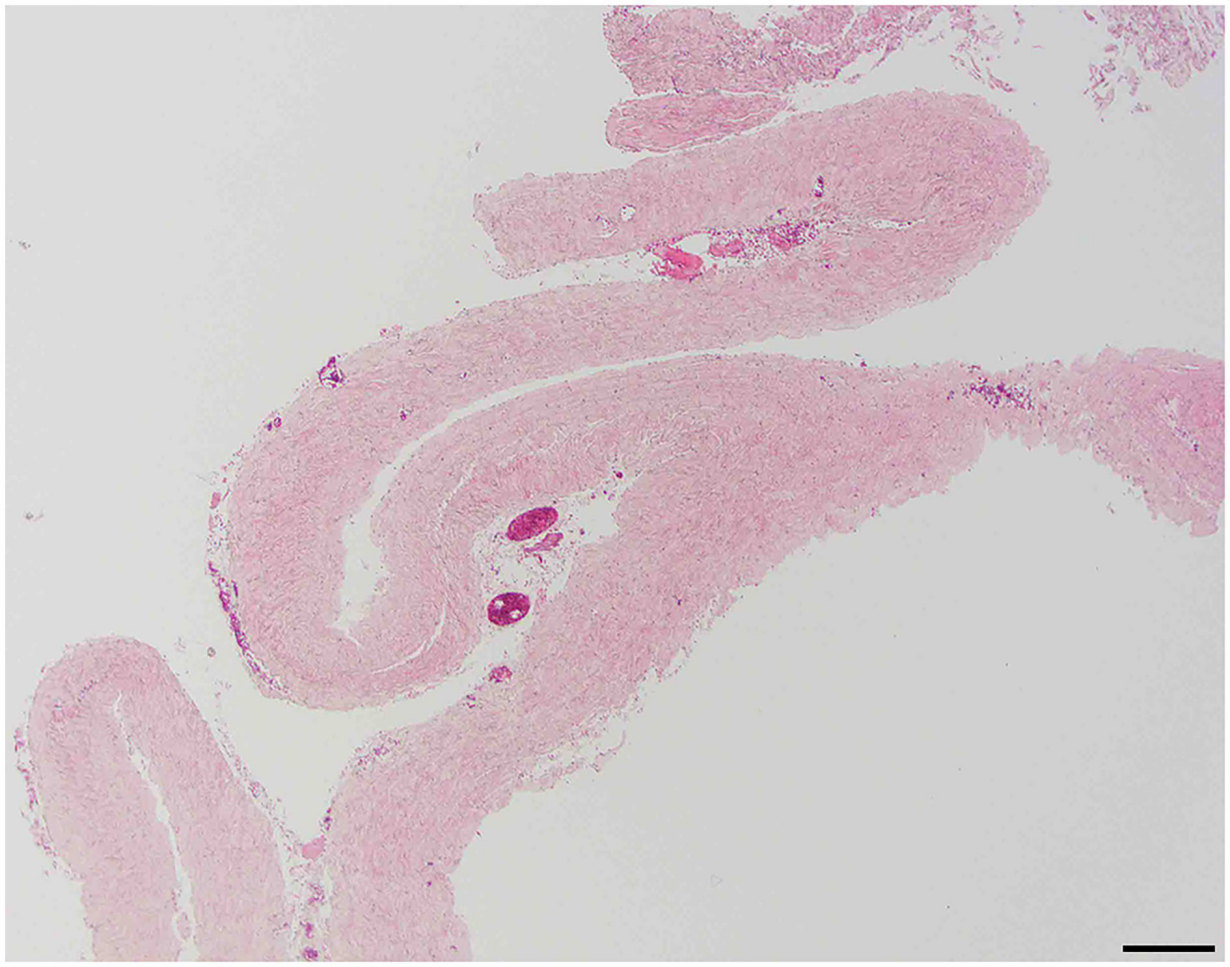
Control specimen; 40 × –Scale bar = 50 microns.

Twelve necropsy samples of dogs who died of causes unrelated to CM were obtained from the Cornell Diagnostic Laboratory from August of 2021 through May of 2022. Of those twelve samples, ten were used to keep the mean age of the control group comparable to that of the group with CM. The average age of the control specimens were 42 months old with a range from 10 to 132 months. All dural samples collected from unaffected dogs were evaluated and interpreted by the same board-certified veterinary pathologist as the previous surgically resected dural samples (ADM). All samples were characterized by a uniform thickness of dural collagen that blended with adjacent, extradural skeletal muscle, adipose, and other soft tissue. Foci of osseous or chondroid metaplasia were not recognized in control samples (Figure 6). Comparably to the samples classified as “no evidence of inflammation or neoplasia,” evidence of haphazard collagen deposition in the dura or excess fibrosis was not observed. In addition to control samples, routine histopathology of the normal canine dura has also been previously described in veterinary literature and texts (44–46). See Table 1 for signalment and cause of death for the ten control dural samples.

**Table 1:** Ten control dural biopsy specimens of dogs that died of causes unrelated to Chiari-like malformation (CM).

**Patient**	**Age**	**Weight (kg)**	**Breed**	**Sex**	**Cause of death**
1	2-year-old	14.3 kg	Pembroke Welsh Corgi	Castrated male	Multicentric peripheral neuroblastoma
2	1.5-year-old	10.3 kg	Beagle	Male Intact	Acute hemorrhagic gastroenteritis
3	1-year-old	9.3 kg	Goldendoodle	Spayed Female	Systemic inflammatory response syndrome
4	5-year-old	8.1 kg	French Bulldog	Spayed Female	Brachycephalic airway syndrome
5	11-year-old	15.7 kg	Beagle	Castrated male	Open (no neurologic disease)
6	10-month-old	22.1 kg	English Bulldog	Intact Male	Open (no neurologic disease)
7	7-year-old	7.4 kg	Mixed breed	Castrated male	Left ventricular cardiomegaly and heart failure
8	11-month-old	31.8 kg	Pitbull terrier	Intact male	Parvovirus
9	10-month-old	33 kg	Labrador Retriever	Intact male	Open (no neurologic disease)
10	5-year-old	25 kg	Basset Hound	Castrated male	Hemopericardium secondary to chemodectoma

### Statistical analysis

The chi-square test was used to analyze associations between histologic diagnosis and categorical variables. For continuous measures, the Kruskal–Wallis non-parametric test was used to compare distributions across pathology categories. Since only four dogs had arachnothelial hyperplasia and three dogs had mineralization, the former category was combined with fibrosis and the latter with osseous metaplasia to form a “collapsed” diagnosis. Based on the unbiased selection of control samples, a Fisher's exact test was performed to compare the prevalence of significant pathology in dogs with CM vs. control dogs. A result was considered statistically significant at the *p* < 0.05 level of significance.

## Results

### Overall prevalence

There were sixty-nine (57.02%) women and fifty-two (42.98%) men. The mean age, duration of pre-surgical clinical signs, and pre-operative QOL were 44.27 months (range 6–132 months), 44.78 weeks, and 2.72 (1–5 scale), respectively. Syringomyelia was found in the cervical region only in 39 of 121 (32.23%) of dogs, in the cervical and thoracic region only in 17 of 121 (14.05%) of dogs, and in the cervical, thoracic, and lumbar region combined in 65 of 121 (53.72%) of dogs. Sixty-six of one hundred twenty-one (54.55%) dural biopsy specimens had histopathology changes; 55 (45.45%) did not. Forty-three of one hundred twenty-one (35.54%) dural biopsy specimens had osseous metaplasia, 16 of 121 (13.22%) had evidence of fibrosis, 4 of 121 (3.31%) had arachnoid hyperplasia, and 3 of 121 (2.48%) had evidence of mineralization.

See Table 2 for a summary of each dural change.

**Table 2:** Histological diagnosis of dura in 121 dogs with surgically corrected CM.

**Diagnosis**	***n* (%)**	**Age at surgery (months)** **mean ±SD**	**Duration of pre-surgery clinical signs (weeks) median (range)**	**Pre-op QOL** **(1-5 scale)** **median (range)**
No inflammation or neoplasia	55 (45.45%)	42.5 ± 24.9	13 (1–312)	3 (1–4)
Osseous metaplasia	43 (35.54%)	42.2 ± 30.7	26 (1–339)	3 (1–4)
Fibrosis	16 (13.22%)	57.1 ± 25.2	20 (1–312)	3 (1–4)
Arachnoid hyperplasia	4 (3.31%)	66.0 ± 34.4	21 (2–104)	3 (2–3)
Mineralization	3 (2.48%)	9.0 ± 6.1	7 (3–9)	3 (2–3)

### Histopathologic classifications

#### No evidence of inflammation or neoplasia

There were 55 of 121 (45.45%) specimens that had no significant findings on dura histopathology. There were 30 of 55 (54.55%) women and 25 of 55 (45.45%) men. The mean age, duration of pre-surgical clinical signs, pre-operative QOL and were 42.45 months, 42.30 weeks, and 2.71 (1–5 scale), respectively. Fifty-five dogs with syringomyelia had normal dura on histology in the following distribution: [cervical region = 15/55 (27.27%), cervical and thoracic region = 10/55 (18.18%), cervical, thoracic & lumbar region = 30/55 (54.55%)]. Normal dural findings were not associated with age, sex, duration of pre-surgical clinical signs, or pre-operative QOL score.

#### Arachnothelial hyperplasia

There were four of one hundred twenty-one specimens (3.30%) that had evidence of arachnothelial hyperplasia on dura histopathology. There were two (50%) women and two (50%) men. The mean age, duration of pre-surgical clinical signs, pre-operative QOL and were 66.00 months, 36.84 weeks, and 2.75 (1–5 scale), respectively. Four dogs who had arachnothelial hyperplasia on histology were also noted to have syringomyelia in the following distribution: [cervical region = 1/4 (25%), cervical and thoracic region = 1/4(25%), cervical, thoracic & lumbar region = 2/4 (50%)].

#### Dural fibrosis

There were 16 of 121 (13.22%) specimens that had evidence of fibrosis on dura histopathology. There were nine (56.25%) women and seven (43.75%) men. The mean age, duration of pre-surgical clinical signs, pre-operative QOL and were 57.13 months, 50.00 weeks, and 2.64 (1–5 scale), respectively. Sixteen dogs with dural fibrosis on histology were also noted to have syringomyelia in the following distribution: [cervical region = 4/16 (25.00%), cervical and thoracic region = 3/16 (18.75%), cervical, thoracic & lumbar region = 9/16 (56.25%)]. Fibrosis was not associated age, sex, duration of pre-surgical clinical signs, or pre-operative QOL score.

#### Dural mineralization

There were 3 of 121 (2.48%) specimens that had evidence of mineralization on dura histopathology. There were two (66.67%) females and one (33.33%) male. The mean age, duration of pre-surgical clinical signs, pre-operative quality of life and were 9.00 months, 6.15 weeks, and 2.67 (1–5 scale) respectively. Three dogs who had dural mineralization on histology were also noted to have syringomyelia in the following distribution: [cervical region = 1/3 (33.33%), cervical and thoracic region = 1/3 (33.33%), cervical, thoracic & lumbar region = 1/3 (33.33%)].

#### Dural osseous metaplasia

There were 43 of 121 (35.53%) specimens that had evidence of osseous metaplasia on dura histopathology. There were 26 (60.47%) females and 17 (39.53%) males. The mean age, duration of pre-surgical clinical signs, pre-operative quality of life and were 42.24 months, 49.45 weeks, and 2.77 (1-5 scale), respectively. Forty-three dogs that had dural osseous metaplasia on histology were also noted to have syringomyelia in the following distribution: [cervical region = 18/43 (41.86%), cervical and thoracic region = 2/43 (4.65%), cervical, thoracic & lumbar region = 23/43 (53.48%)]. Osseous metaplasia with was not associated with age, sex, duration of pre-surgical clinical signs, or pre-operative QOL.

#### Control specimens

Sixty-six of one hundred twenty-one (54.55%) dural biopsy specimens had histopathology changes in dogs with CM; 55 (45.45%) did not. Zero of 10 dogs in the control samples (0%) had histopathology changes. The *p*-value for this result is <0.0006.

### Location of syrinx

Syrinx location relative to histopathologic classifications is summarized in Table 3.

**Table 3:** Histological diagnosis of dura in 121 dogs and associated syrinx location.

	**Histopathologic diagnosis**					
**Syrnix** **location**	**No evidence of inflammation or neoplasia**	**Arachnoid hyperplasia**	**Fibrosis**	**Mineralization**	**Osseous metaplasia**	**Total**
Cervical	15	1	4	1	18	39
Cervical and thoracic	10	1	3	1	2	17
Cervical and thoracic and lumbar	30	2	9	1	23	65
Total	55	4	16	3	43	**121**

Bold number indicates total number of study dogs.

## Discussion

To the authors' knowledge, this is the first study to describe dural histopathologic changes at the craniocervical junction in CKCS with CM. In this study, we found that dural histopathology could be classified into five categories based on the predominant histopathological feature (i.e., normal dura, arachnothelial hyperplasia, fibrosis, mineralization, and osseous metaplasia). We found pathology in dura in slightly more than half of dogs that underwent a FMD with cranioplasty and durectomy for CM. Of particular interest, dural changes were observed in dogs with only 4 weeks of pre-surgical clinical signs as noted by the owners at presentation.

According to some, disturbances in normal CSF flow, including the formation of high velocity jets, at the craniocervical junction have been reported in human and veterinary patients may be the cause of the dural changes observed due to physical insult to the surrounding tissues (47–50). The phenomenon called the Venturi effect has been proposed to be involved in syrinx formation. The Venturi effect is based on the phenomenon of a jet of cerebrospinal fluid flowing from higher to lower velocity leading to the spinal cord substance being pulled in an outward direction, facilitating the accumulation of fluid in the syrinx cavity. The Venturi effect has been theorized to be responsible for the formation of syringomyelia in veterinary patients (12, 51). Others note that the pathophysiology may be related to nervous system pistoning into the foramen magnum causing secondary changes in pressure and overall dural compliance (18, 23, 52). The consequence of histologic changes as it relates to CSF flow is unknown at this time, but changes in tissue pliability may impact the movement of CSF. Future biomechanical studies evaluating dural compliance should be considered.

Age at the time of decompression was not found to be associated with the various changes observed on histopathology. Although age matching of the control samples was not performed due to the retrospective nature of this study, the average age of the control specimen group was comparable to those of dogs with CM in the current study (mean = 42 months for controls; mean = 44.27 for dogs with CM). The presumption that older dogs may have more chronic changes was not supported in the study. This may depend on the age of manifestation of coning, decompensation of herniation, vascular compromise, and/or syrinx formation, however, the small sample size in some of the classifications may have had an impact on statistical analysis.

The syrinx location is consistent with the author's observations in over 350 dogs with CM. Others have reported similar findings (12, 22, 53–57). The presumption that dogs with more distant syringomyelia (involving cervical, thoracic, and lumbar regions) may have more chronic histopathological changes was not supported in the study; however, the small sample size in some of the classifications may have had an impact on statistical analysis.

Pre-operative QOL score was not found to be associated with any of the previously mentioned histopathologic changes. The severity of clinical signs, namely pain and phantom scratching, has been attributed to asymmetrical dorsal horn involvement and brachycephaly (22, 23, 58–60), while neck pain may be directly related to the constriction at the craniocervical junction (61, 62). The presumption that dogs with more pain and, therefore, a lower QOL score may have more constriction at the cervicomedullary junction due to more significant changes in the dura in the cervicomedullary junction was not supported in the study; however, the small sample size in some of the classifications may have had an impact on statistical analysis.

The authors found no reports of histopathologic findings of full-thickness dura mater in human patients with Chiari malformation having FMD. One study evaluated histopathology of the outer layer of the dura (inner layer and subdural space remain intact) in CM-I patients with SM and compared them to control autopsy specimens (39). The thickness of the resected dural bands varied from 3–5 mm, which was noted to be three to five times thicker than that of an autopsy specimen. The histopathologic examination focused on the regularity of the collagen arrangements within the dura. Sections of dura examined from CM-I patients with SM showed an irregular collagen pattern and showed degenerative changes such as hyalinosis, calcification, and/or ossification. All eight specimens were noted to have irregular collagen arrangements and hyalinosis. One specimen had evidence of calcification only, three showed ossification only, and four showed both calcification and ossification. There were no calcification or ossification noted in any of the control autopsy specimens (39). Because of the nature of how the dura samples in the current study were harvested and processed, fiber orientation examination in transverse and longitudinal sections was not possible, making this a limitation of the current data. Nakamura et al. concluded that the thickened dura observed on histopathologic examination seemed to be due to a chronic state and presumed this to be a sequela of dissociation in CSF pressure between the cranial cavity and spinal cord with increased pressure at the craniocervical junction.

The previous studies in dogs with CM have proposed that the concurrent diagnosis of syringomyelia occurs from altered CSF flow at the level of the foramen magnum (8, 12, 30). The objective of surgery in both human and veterinary medicine is the restoration of normal CSF flow, by decompression which includes the removal of bone and in the opinion of some, the dura. There remains significant debate regarding the merits of osseous decompression alone (leaving the dura intact) vs. osseous and soft tissue decompression (removing the outer layer of the dura and performing a duraplasty) vs. osseous decompression and soft tissue decompression (full thickness durotomy and addressing arachnoid pathology) (26, 31–34). Meningeal pathology has been suspected to play a role in the pathophysiology of CM-I and subsequent syrinx formation in humans (63–65). Tubbs et al. were the first to report arachnoid pathology, also termed arachnoid veils and/or adhesions, at the foramen Magendie in humans. The finding of arachnoid pathology was significantly more common in CM-1 patients with SM than in those without.

Although we note the majority of dogs had dural pathology present at the time of decompression, we do not yet know the significance of this pathology as it relates to the development of SM. Since dogs do not have a foramen Magendie, and this area is normally “sealed” by the velum, it is uncertain whether the aforementioned findings are relevant. All dogs with CM at our practice undergo a full thickness durectomy at the time of surgery, making it difficult to assess the effectiveness of leaving the dura intact (control group of dogs with osseous decompression only, without durectomy). We do not know the implications of leaving the dura intact or what the previously described histopathologic changes have on clinical outcomes.

### Limitations of the study

Attributable to the nature of how samples were harvested and processed, fiber orientation examination in transverse and longitudinal sections was not possible and considered a limitation of the samples evaluated in this study. Future and more advanced prospective studies should include marking the rostral and caudal boundaries of the durectomy for orientation purposes, developing specialized stains for collagen subtypes, elastic fibers and reticulin, and to consider the use of polarized light microscopy for further sample evaluation. Our QOL scores are subjective and pre-operative owner assessments of the patient are considered a limitation of the current retrospective study. Future studies could use a more detailed and specific pain scoring system (66).

## Conclusions

Varying classifications of dural and histopathology were observed in a majority of CKCS with CM having FMD with cranioplasty. These dural changes can be observed in dogs with only 4 weeks of pre-surgical clinical signs. No association was found between the different histopathologic classifications and age, duration of clinical signs, syrinx location, and the QOL before surgery. Dural changes at craniocervical junction have been insufficiently investigated in CM-affected dogs despite the high prevalence in CKCS and a plethora of published studies that suggest meningeal structural abnormalities based on imaging findings. Future studies evaluating CSF flow before and after decompression, as well as biomechanical testing of abnormal resected dura could be performed in order to assess structural compliance of the dura and if the dura plays a role in altering CSF flow at the level of the foramen magnum. Evaluation of the degree and severity of pre- and post-operative neurological clinical signs and MRI changes in comparison to dura histopathologic changes should also be considered.

## Data availability statement

The original contributions presented in the study are included in the article/supplementary material, further inquiries can be directed to the corresponding author.

## Ethics statement

The animal study was reviewed and approved by Long Island Veterinary Specialists Ethics Committee. Written informed consent was obtained from the owners for the participation of their animals in this study.

## Author contributions

JH, DM, and CL were the major contributors to the writing of the manuscript. JS, MO'D, and ML contributed the statistical analysis of all histopathologic data in the manuscript. AM reviewed all the histopathologic specimens. All authors read and approved the final manuscript.

## Conflict of interest

The authors declare that the research was conducted in the absence of any commercial or financial relationships that could be construed as a potential conflict of interest.

## Publisher's note

All claims expressed in this article are solely those of the authors and do not necessarily represent those of their affiliated organizations, or those of the publisher, the editors and the reviewers. Any product that may be evaluated in this article, or claim that may be made by its manufacturer, is not guaranteed or endorsed by the publisher.
